# Tissue-specific signatures in tick cell line MS profiles

**DOI:** 10.1186/s13071-019-3460-5

**Published:** 2019-05-06

**Authors:** Dmitry S. Loginov, Yana F. Loginova, Filip Dycka, Katharina Böttinger, Pavlina Vechtova, Jan Sterba

**Affiliations:** 1 Faculty of Science, University of South Bohemia, Branišovská 1760, 37005 Ceske Budejovice, Czech Republic; 2 Institute of Parasitology, Biology Centre of the Czech Academy of Sciences, Branišovská 1760, 37005 Ceske Budejovice, Czech Republic; 3 Orekhovich Institute of Biomedical Chemistry, Pogodinskaja str. 10, Moscow, 119191 Russia

**Keywords:** Tick cell line, Tick organs, Tick, Biotyping, MALDI-TOF MS

## Abstract

**Background:**

The availability of tick *in vitro* cell culture systems has facilitated many aspects of tick research, including proteomics. However, certain cell lines have shown a tissue-specific response to infection. Thus, a more thorough characterization of tick cell lines is necessary. Proteomic comparative studies of various tick cell lines will contribute to more efficient application of tick cell lines as model systems for investigation of host-vector-pathogen interactions.

**Results:**

Three cell lines obtained from a hard tick, *Ixodes ricinus*, and two from *I. scapularis* were investigated. A cell mass spectrometry approach (MALDI-TOF MS) was applied, as well as classical proteomic workflows. Using PCA, tick cell line MS profiles were grouped into three clusters comprising IRE/CTVM19 and ISE18, IRE11 and IRE/CTVM20, and ISE6 cell lines. Two other approaches confirmed the results of PCA: in-solution digestion followed by nanoLC-ESI-Q-TOF MS/MS and 2D electrophoresis. The comparison of MS spectra of the cell lines and *I. ricinus* tick organs revealed 29 shared peaks. Of these, five were specific for ovaries, three each for gut and salivary glands, and one for Malpighian tubules. For the first time, characteristic peaks in MS profiles of tick cell lines were assigned to proteins identified in acidic extracts of corresponding cell lines.

**Conclusions:**

Several organ-specific MS signals were revealed in the profiles of tick cell lines.

**Electronic supplementary material:**

The online version of this article (10.1186/s13071-019-3460-5) contains supplementary material, which is available to authorized users.

## Background

Ticks are hematophagous ectoparasites which infest every class of vertebrates, including mammals, birds, reptiles, etc. Ticks are the second most common arthropod pathogen vector after mosquitoes [1], but from the viewpoint of transmission of different pathogenic agents, they are taking first place [2]. Ticks transmit fungi, bacteria, viruses and protozoa causing many diseases, such as Lyme disease, tick-borne encephalitis, human granulocytic anaplasmosis and louping ill [2]. Currently, there is a growing global threat of emerging or re-emerging tick-borne diseases. Due to climate change, ticks are forced to expand their geographical distribution and, subsequently, the distribution of tick-transmitted pathogens is increasing [3].

Nowadays, different studies concerning ticks are facilitated by the availability of *in vitro* cell culture systems. Tick cell lines are very useful tools in defining the complex nature of the host-vector-pathogen interactions [4]. They can survive for long periods with minimal attention compared to mammalian cell lines that makes them suitable for studies on virus persistence and propagation, which requires prolonged incubation times [5]. Another benefit of their use is the possibility to study tissue-specific responses resulting from the embryonic origin of tick cell lines [4]. Nevertheless, the main drawback of using tick cell lines as a model for investigation of vector-pathogen interactions is their phenotypical and genotypical heterogeneity. Thus, tick cell lines obtained even from the same tick species may differently respond to infection [6–9]. To overcome this obstacle, comparative studies of tick cell lines, including proteomic studies, are required.

Matrix-assisted laser desorption/ionization time-of-flight mass spectrometry (MALDI-TOF MS) has emerged as a reliable method for the identification and characterization of diverse microorganisms [10]. This approach, utilizing peptide/protein desorption and ionization from the cells, provides a simple diagnostic tool based on unique mass spectra of the analyzed samples. MALDI-TOF MS has been already successfully applied for tick species identification [11], characterization of tick developmental stages [12] and detection of pathogens in ticks [13].

In the present study, three cell lines originating from *Ixodes ricinus* (IRE11, IRE/CTVM19, IRE/CTVM20) and two from *I. scapularis* (ISE6, ISE18) ticks were used. The comparison of their MS profiles between each other and tick organs was performed to understand the nature of tick cell lines better. Several characteristic MS signals were assigned with proteins extracted under conditions used for MALDI-TOF MS experiments. The results of MS profiling of tick cell lines were verified by two-dimensional gel electrophoresis.

## Results

**Fig. 1:**
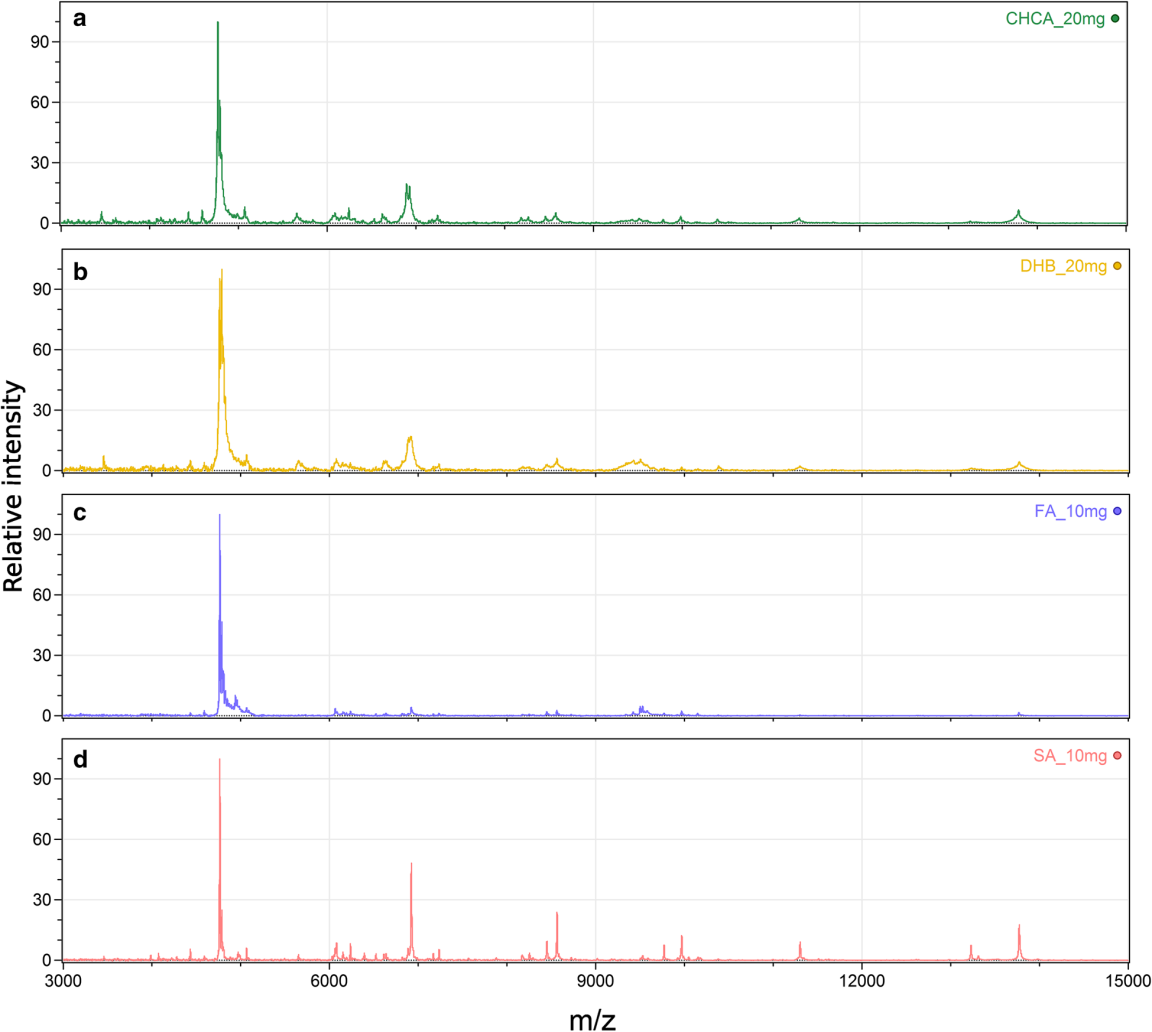
Positive ion MALDI-TOF mass spectra of the IRE/CTVM19 tick cell line. Matrices applied by the dried droplet method were: **a** CHCA (20 mg/ml), **b** DHB (20 mg/ml), **c** FA (10 mg/ml), and **d** SA (10 mg/ml) in acetonitrile/2.5% v/v trifluoroacetic acid, 7:3 (v/v)

Optimization of MS profiling conditions was carried out using the IRE/CTVM19 tick cell line. Four matrices, namely CHCA, DHB, FA, and SA, were used to acquire MALDI MS spectra (Fig. 1). The MS profiles obtained using CHCA or DHB as a matrix were very similar with well resolved low molecular mass peaks (m/z < 9000). A higher number of peaks were detected with FA matrix, but their intensities were low. SA allowed obtaining spectra containing well-defined peaks also at higher m/z values (> 9000).

**Fig. 2:**
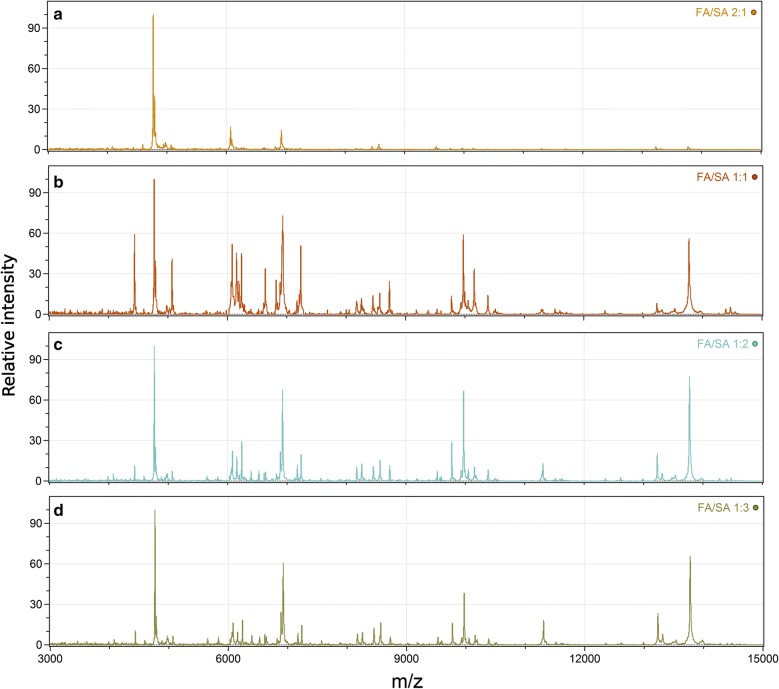
Combining matrix compounds to enhance the signal intensity and the number of peaks in peptide/protein profiles. MS profiles of the IRE/CTVM19 tick cell line were obtained after mixing FA and SA in ratios of: **a** 10:5, **b** 5:5, **c** 5:10 and **d** 5:15 mg/ml

To improve the mass spectral information content, the MS profiles of tick cell lines were measured with combinations of FA and SA matrices at different ratios (Fig. 2). Matrices were mixed in FA:SA ratios of 10:5, 5:5, 5:10 and 5:15 mg/ml. The best spectra in terms of signal-to-noise ratio and number of peaks were obtained using the FA:SA mixtures with concentrations of 5:10 and 5:15 mg/ml, with the highest number of peaks detected for the first composition. Thus, the 5:10 mg/ml FA:SA mixture was used in all further MS profiling experiments.

### Comparison of MALDI-TOF MS spectra

**Fig. 3:**
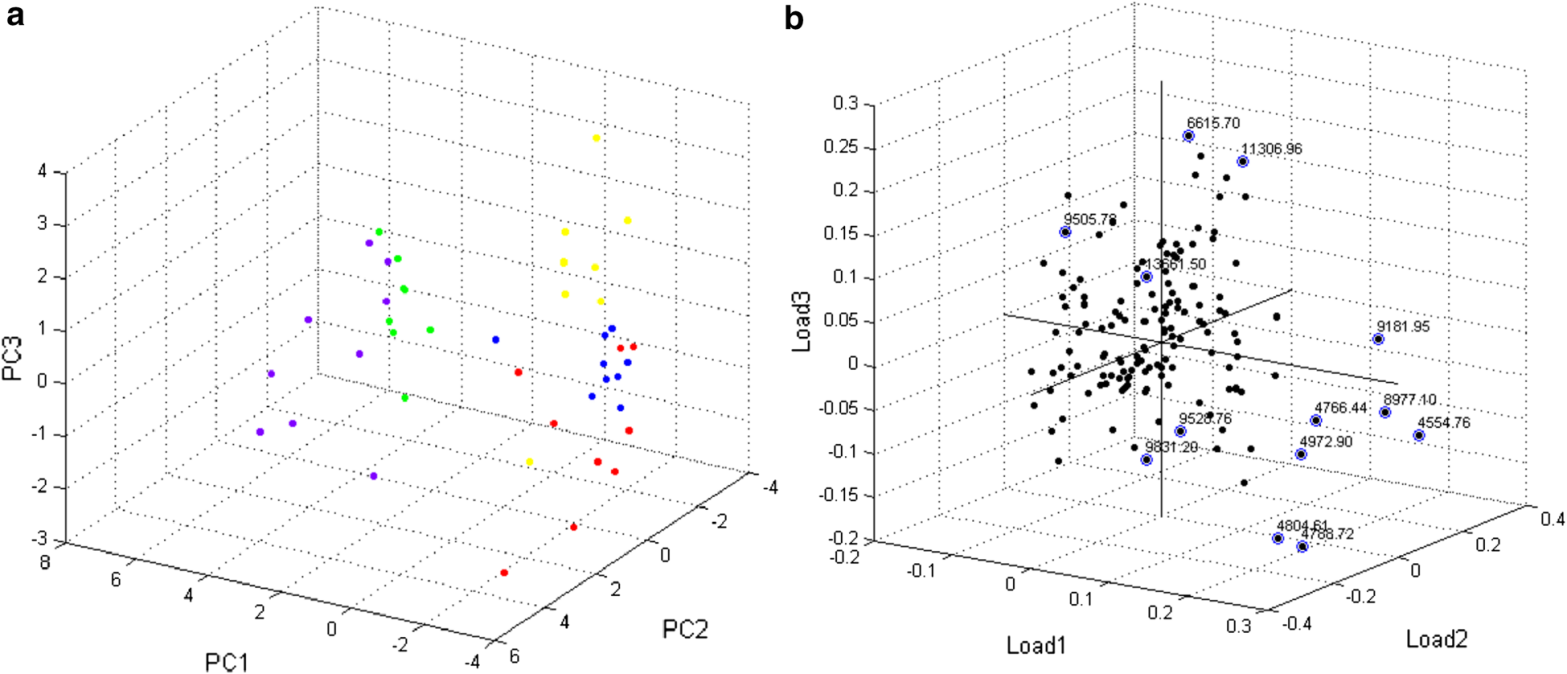
Principal component analysis from the MS spectra of tick cell lines. **a** PCA three-dimensional plot from MS spectra of tick cell lines: IRE11 (red dots); IRE/CTVM19 (green dots); IRE/CTVM20 (blue dots); ISE6 (yellow dots); and ISE18 (purple dots). MS profiles of tick cell lines formed three clusters: IRE/CTVM19 and ISE18, IRE11 and IRE/CTVM20, and ISE6. **b** 3D factor loadings plot. Factors with loadings > 0.2 are marked in blue

The resultant MS profiles of tick cell lines were compared between each other, and with MS spectra of tick organs. Principle component analysis (PCA) was performed to visualize their distribution. Figure 3 represents the PCA of tick cell line profiles. The dot-reflecting MS spectra formed three clusters. IRE/CTVM19 cells were grouped with ISE18 and IRE11 with IRE/CTVM20. The MS profiles of the ISE6 cell line were defined in its own group (Fig. 3a).

**Fig. 4:**
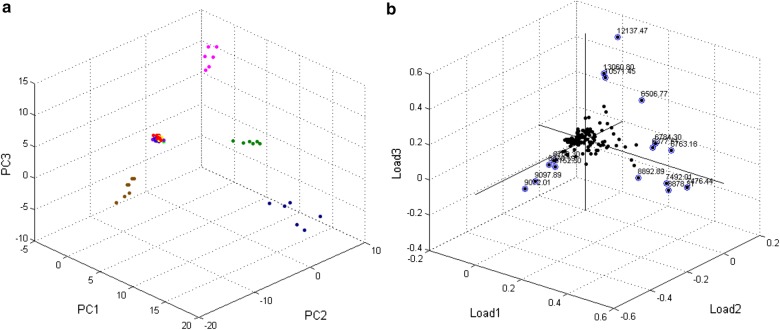
Principal component analysis from the MS spectra of tick cell lines and tick organs. **a** PCA three-dimensional image from MS spectra of tick cell lines and tick organs: IRE11 (red dots); IRE/CTVM19 (light green dots); IRE/CTVM20 (light blue dots); ISE6 (yellow dots); ISE18 (purple dots); gut (deep green); salivary glands (brown); ovaries (magenta); and Malpighian tubules (deep blue). Distinct clusters were formed by MS profiles of tick cell lines and organs. **b** 3D factor loadings plot. Factors with loadings > 0.2 are marked in blue

Factors with a load > 0.2 were selected as the most contributing to the separation of tick cell lines MS profiles (Table 1). A comparison of tick cell lines with MS profiles of tick organs showed clear clustering of spectra (Fig. 4a).

**Table 1:** List of peaks with the highest influence (load > 0.2) on the clustering of tick cell line MS profiles

Load	m/z	IRE11	IRE/CTVM19	IRE/CTVM20	ISE6	ISE18
1	4554	−	−	−	−	+
	4766	+	+	+	+	+
	4788	+	+	+	+	+
	4804	−	+	+	−	+
	4972	−	+	−	−	+
	8977	−	+	−	−	+
	9182	−	+	−	−	+
2	9506	+	+	+	+	+
	9528	−	+	−	−	+
	9831	+	+	+	+	+
3	6615	+	+	+	+	+
	11306	+	+	+	+	+

Key: −, not detected; +, detected

The profiles of tick cell lines were grouped separately from all tested organs. The load plots allowed selection of the most important MS peaks for clustering (Fig. 4b). Signals with m/z 8478, 8708, 8793, 9072, 9097 and 9152 discriminated the salivary gland spectra; m/z 10571, 12137 and 13060 the ovary spectra; m/z 7476, 7492 and 8878 the Malpighian tubule spectra; and m/z 6377, 6784 and 6793 the gut spectra.

The qualitative comparison of all MS profiles was performed using mMass software with 500 ppm tolerance. For this purpose, average spectra were built for each sample. Overall, 18, 17, 15 and 11 peaks in the MS profiles of tick cell lines were matched by peaks from ovary, Malpighian tubule, salivary gland and gut spectra, respectively, which resulted in the total assignment of 29 peaks (Table 2). Out of them, five peaks with m/z 4360, 4583, 6068, 6822 and 9971 were present only in ovaries, three each for the gut (m/z 4788, 6844 and 8451) and salivary glands (m/z 4210, 6286 and 9182), and one with m/z 9506 was present in Malpighian tubules. Four peaks, observed in organ spectra, with m/z 6068, 6822, 9506 and 9971 were common for all tick cell lines.

**Table 2:** List of shared peaks between tick cell line and tick organ MS profiles

Peak (m/z)	IRE11	IRE/CTVM19	IRE/CTVM20	ISE6	ISE18	G	SG	Ov	MT
3923	+	+	+	+	+	−	+	+	−
4210	+	−	+	+	−	−	+	−	−
4360	+	−	+	+	−	−	−	+	−
4583	+	−	+	-	−	−	−	+	−
4766	+	+	+	+	+	−	+	+	+
4788	+	+	+	+	+	+	−	−	−
6034	−	+	−	+	−	−	+	+	+
6068	+	+	+	+	+	−	−	+	−
6154	+	+	+	+	+	+	+	+	+
6238	+	+	+	+	+	+	+	+	+
6286	+	+	+	+	−	−	+	−	−
6527	+	+	+	+	+	+	+	+	+
6822	+	+	+	+	+	−	−	+	−
6844	+	+	+	+	−	+	−	−	−
6936	+	−	+	+	−	+	−	+	+
8172	+	+	+	+	+	+	−	+	+
8277	+	−	−	+	−	−	+	−	+
8451	+	+	+	+	+	+	−	−	−
8565	+	+	+	+	+	+	+	+	+
8728	+	+	+	+	+	+	−	+	+
9182	−	+	−	−	+	−	+	−	−
9506	+	+	+	+	+	−	−	−	+
9590	+	+	+	+	+	−	−	+	+
9971	+	+	+	+	+	−	−	+	−
11306	+	+	+	+	+	−	+	−	+
13231	+	+	+	+	+	−	+	+	+
13312	+	+	+	+	+	−	+	−	+
13521	−	+	−	−	−	+	−	−	+
13775	+	+	+	+	+	−	+	+	+
Total	26	24	25	26	20	11	17	18	15

*Abbreviations*: G, gut; SG, salivary glands; Ov, ovaries; MT, Malpighian tubules

*Key*: −, not detected; +, detected

Three peaks relevant for the clustering of tick cell lines as described above were found in the spectra of tick organs. The peak with m/z 4766 was absent only in the gut spectra, the peak with m/z 11306 was observed in the salivary gland and Malpighian tubule spectra, and the peak with m/z 4788 was detected only in the gut spectra. Additionally, four peaks with m/z 6154, 6238, 6527 and 8565 were present in all tested samples. In contrast, none of the MS signals with the highest influence onto the organ clustering were observed in the spectra of tick cell lines.

### Identification of proteins contributing to the signals detected in MS profiles of tick cell lines

**Fig. 5:**
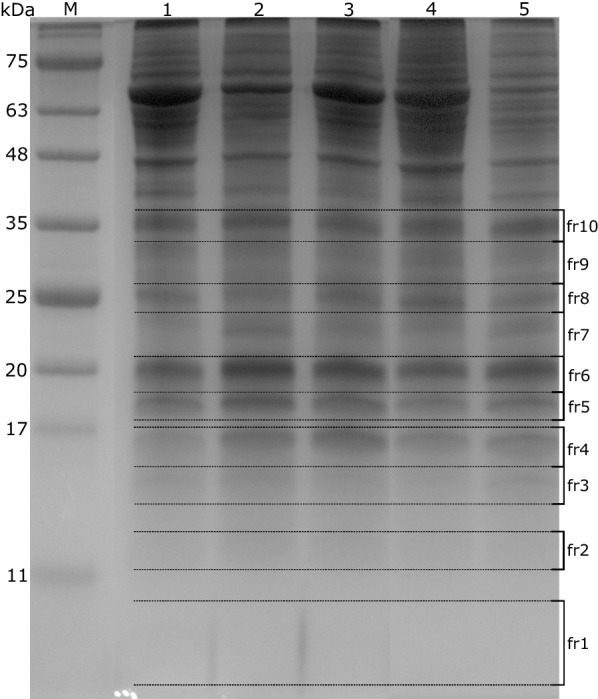
Tricine-SDS-PAGE gel with separated peptide/protein ACN/TFA extracts from tick cell lines. Lane M: marker; Lane 1: IRE11; Lane 2: IRE/CTVM19; Lane 3: IRE/CTVM20; Lane 4: ISE6; Lane 5: ISE18. Numbered gel segments (labeled as fr) were processed for in-gel digestion and further analyses

Tick cell line proteins released upon ACN/2.5% TFA (v/v ratio 7:3) treatment were separated by Tricine-SDS-PAGE to better resolve the low molecular weight region (Fig. 5).

The gel was cut into the fractions as depicted in Fig. 5. All fractions were subjected to in-gel digestion with subsequent peptide separation using a microgradient column. Proteins were identified by MALDI-TOF/TOF MS/MS (Additional file 1: Table S1). As a result, two peaks with m/z 8565 and 9773, common for all tested cell lines, were assigned to the putative cytoplasmic cystatin (UniProt ID: A0A131Y5V6) and putative ubiquitin/40S ribosomal protein S27a fusion (UniProt ID: A0A0K8RPM6), respectively, based on an agreement in the molecular mass. A tolerance of ± 50 Da was accepted for an assignment.

To increase the number of annotated peaks, the protein acidic extracts of tick cell lines were subjected to in-solution digestion followed by nanoLC-ESI-Q-TOF MS/MS. Nine more peaks from tick cell line MS profiles were assigned to proteins (Table 3).

**Table 3:** Assigned characteristic peaks in the MS profiles of *Ixodes* spp. tick cell lines to proteins identified in the corresponding extracts

m/z	Accession^a^	Description^b^	Species	MW (Da)^c^	Tick cell line^d^	Tick organ^e^
6154	B7QEH2	Conserved hypothetical protein (F1-ATPase epsilon superfamily)	*I. scapularis*	6160	All	All
6656	Q4PM47	Ribosomal protein S29	*I. scapularis*	6653	IRE/CTVM19; ISE6; ISE18	–
8565	A0A131Y5V6	Putative cytoplasmic cystatin	*I. ricinus*	8612	All	All
9182	V5H067	Putative ciboulot_ partial	*I. ricinus*	9189	IRE/CTVM19; ISE18	SG
9773	A0A0K8RPM6	Putative ubiquitin/40s ribosomal protein s27a fusion	*I. ricinus*	9696	All	–
9971	V5H1M0	Putative ATP synthase e chain *Rhipicephalus sanguineu*s ATP synthase e chain	*I. ricinus*	9964	IRE11; IRE/CTVM19; IRE/CTVM20	Ov
	Q4PM77	ATP synthase E chain	*I. scapularis*		ISE6; ISE18	
11515	A0A0K8RQR8	Putative histone h4_partial	*I. ricinus*	11488	IRE/CTVM20	–
13312	A0A0K8RIJ1	Putative histone 2a	*I. ricinus*	13355	IRE11; IRE/CTVM19; IRE/CTVM20	SG, MT
	B7PSI5	Histone 2A	*I. scapularis*		ISE6; ISE18	
13521	A0A090XDF4	Putative glycine-rich secreted protein 95	*I. ricinus*	13504	IRE/CTVM19	G, MT
13775	A0A0K8RB64	Putative histone h2b	*I. ricinus*	13810	IRE11; IRE/CTVM19; IRE/CTVM20	SG, Ov, MT
	Q4PM63	Histone 2B	*I. scapularis*		ISE6; ISE18	
15364	A0A0K8R3K9	Putative histones h3 and h4	*I. ricinus*	15318	IRE/CTVM19	–

^a^Accession number refers to the sequence retrieved from the UniProt protein database

^b^Description of the respective protein accession. Protein homologs were assigned to unknown proteins *via* BLAST search

^c^Theoretical molecular weight

^d^Presence of a peak in the MS profiles of tick cell lines

^e^Presence of a peak in the MS profiles of tick organs

*Abbreviations*: G, gut; SG, salivary gland; Ov, ovary; MT, Malpighian tubules

Out of the 11 assigned MS peaks, six with m/z 6154, 8565, 9773, 9971, 13312 and 13775 were present in the MS profiles of all tested tick cell lines. The peak with m/z 11515 was found only in the spectra of the IRE/CTVM20 cell line, and two peaks with m/z 13521 and 15364 were characteristic for the IRE/CTVM19 cell line. Two signals with m/z 6154 and 8565, detected in all spectra including tested organs, were assigned to conserved hypothetical protein (UniProt ID: B7QEH2) and putative cytoplasmic cystatin (UniProt ID: A0A131Y5V6), respectively (Table 3).

A total of 49, 49, 71, 46 and 101 proteins were identified in the acidic extracts of IRE11, IRE/CTVM19, IRE/CTVM20, ISE6 and ISE18 tick cell lines, respectively (Additional file 2: Tables S2–S6). Protein profiles were compared separately for *I. ricinus* and *I. scapularis* derived cell lines. In the case of *I. ricinus* cells, 17 proteins were present in all samples, including six histones and two ribosomal proteins. IRE11 and IRE/CTVM20 cells shared 17 proteins, whereas only two and eight proteins were common for these cell lines and IRE/CTVM19, respectively (Additional file 3: Figure S1a). The highest number of shared proteins between IRE/CTVM19 and the other cell lines was found for ISE18: 27 proteins (Additional file 3: Figure S1b). These data support our results of tick cell line MS profiling.

The *I. scapularis* cell lines, ISE6 and ISE18, shared 35 proteins. Of these, the major protein groups were histones (10 proteins) and ribosomal proteins (four proteins), similarly to the *I. ricinus* cells (Additional file 3: Figure S1b).

### 2D electrophoresis of tick cell line protein extracts

**Fig. 6:**
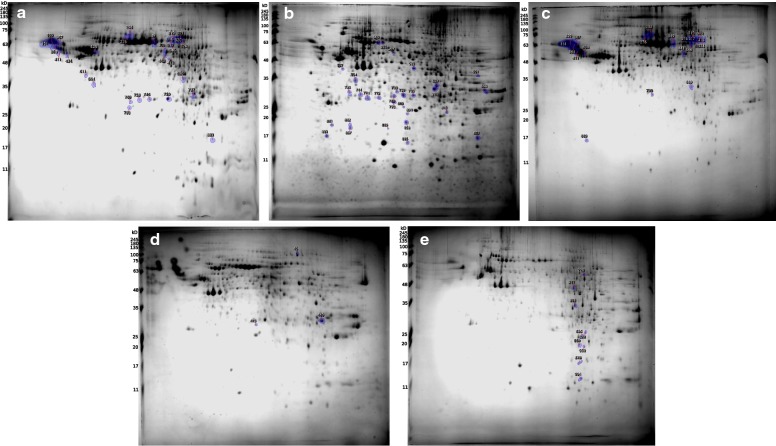
Two-dimensional electrophoresis of tick cell line protein extracts. **a** IRE11. **b** IRE/CTVM19. **c** IRE/CTVM20. **d** ISE6. **e** ISE18. Gels were compared within IRE and ISE groups using the SameSpots software, and protein spots with significantly different expression (ANOVA, *P* < 0.05) were excised from the gels for MALDI-TOF/TOF MS/MS (marked in blue). Protein ladder: 245, 180, 135, 100, 75, 63, 48, 35, 25, 20, 17, 11 kDa

Proteomic maps of tick cell line protein extracts were compared using the SameSpots software. Cell lines were divided into two groups based on their origin: derived from *I. ricinus* and *I. scapularis* ticks. The number of detected spots was 760 and 559 for IRE and ISE cells, respectively. Protein profiles of cell lines were very similar within these two groups (Fig. 6).

Nevertheless, some differently expressed proteins were detected. In the present study, we focused on the identification of qualitatively different protein spots. These spots were excised and processed for MALDI-TOF/TOF MS/MS analysis as described. Overall, 55 spots were cut from the IRE 2D gels, including six, 21 and one unique protein spots for IRE11, IRE/CTVM19 and IRE/CTVM20 cell lines, respectively. Out of them, 17 spots were shared by IRE11 and IRE/CTVM20 cells. In accordance with previous results, these cell lines were less similar to IRE/CTVM19: the number of the shared spots was seven and three for IRE11 and IRE/CTVM20, respectively. In the case of ISE cell lines, nine spots were cut for ISE18 cells, and three for ISE6. Overall, 26 and five proteins were identified for IRE and ISE cell lines, respectively (Additional file 4: Table S7).

## Discussion

MALDI-TOF MS is a powerful tool for cell characterization and identification of different organisms. It facilitates cell clustering based on their unique mass spectrometric profiles (fingerprints). In the present study, this method was used to study tick cell lines for the first time.

All cell lines were cultivated at 28 °C to minimize the differences in MS profiles caused by various cultivation conditions; furthermore, lower temperature provides conditions with more resemblance to the off-host tick environment, which also allows a comparison with the unfed tick tissue spectra.

For a proper cell characterization, it is essential to obtain spectra with well-resolved peaks in an m/z range of 1000–20,000. One of the factors affecting MS signal quality is the matrix used in the study. Often, the matrix of choice for identification of arthropod vectors and pathogens they transmit is CHCA [14]. To our knowledge, there have been no reports related to the optimization of matrix composition for the MS profiling of ticks. Thus, we compared four matrices using the IRE/CTVM19 tick cell line. MS profiles acquired with CHCA and DHB matrices showed MS peaks with m/z > 9000 with low resolution. This is not surprising as these matrices are commonly used for identification of peptides or glycans. When using SA as a matrix, spectra with well-resolved peaks even at higher m/z were obtained, in accordance with literature data [15], but some low molecular mass peaks were lost. Thus, combinations of FA and SA matrices were applied for the MS profiling of tick cell lines [15]. The highest mass spectral information content was obtained with FA and SA mixed in a ratio of 5:10 mg/ml.

Submission of tick cell line MS profiles to PCA revealed the formation of three clusters (Fig. 2a). Surprisingly, cell lines IRE/CTVM19 and ISE18, obtained from different tick species, were clustered together, and ISE6 cells were distinct from all other tested cell lines. Peaks with m/z < 10,000 played the main role in the discrimination of tick cell lines.

At the present moment, the phenotypic nature of tick cell lines is unclear. For example, the response of ISE6 cells to *Anaplasma phagocytophilum* infection resembled that of tick hemocytes at the transcriptomic level, whilst IRE/CTVM20 reacted in the same way as tick midguts [6]. Thus, we compared MS profiles of tick cell lines with those obtained from tick organs. The idea behind this was to find out some tissue-specific signatures in the MS spectra of cells. Although cell profiles were distinctly clustered separately from all tested organs (Fig. 4a), the origin of several peaks was determined (Table 2). Nevertheless, none of the peaks with the highest influence on the discrimination of tick organs was found in the cell line spectra, meaning no clear similarity of cell lines to any of the tested organs.

In addition to the characterization of different organisms by MALDI-TOF MS, the acquired MS profiles contain valuable information related to the identification of possible protein markers for tick cell lines or organs [16]. To identify the proteins contributing to the MS spectra of tick cell lines, their acidic protein extracts were both separated by Tricine-SDS-PAGE for MALDI-TOF/TOF MS/MS of protein bands with molecular masses up to 35 kDa (Fig. 5), and in-solution digested followed by nanoLC-ESI-Q-TOF MS/MS. Overall, 11 peaks from cell line MS profiles were assigned to proteins. Four signals with m/z 11515, 13312, 13775 and 15364 were matched with different histone proteins, and two (m/z 6656 and 9773) with ribosomal proteins. These two groups of proteins are well-known markers for biotyping and identification [16]. However, the proteins most promising as possible markers for tick identification were the conserved hypothetical protein (UniProt ID: B7QEH2, m/z 6154) and the cytoplasmic cystatin (UniProt ID: A0A131Y5V6, m/z 8565), as they were assigned to the signals found in the spectra of all samples, including tick organs.

Comparison of protein lists of acidic extracts and 2D proteomic maps of *I. ricinus* cell lines confirmed results of MS profiling: IRE/CTVM19 cells were the least similar to others. Of all the identified differentially expressed *I. ricinus* proteins, four were related to the cytoskeleton formation (Additional file 4: Table S7), which indicates some differences in its structure between cell lines. Additionally, proteomic profiles of cell lines varied in the set of protein isoforms, e.g. putative flavonol reductase/cinnamoyl-CoA reductase (UniProt ID: A0A147BUB5), or putative nucleoside diphosphate-sugar hydrolase of the mutt nudix family (UniProt ID: A0A131Y0B8). The existence of protein isoforms might be related to the differences in metabolic processes caused by a tissue-specific proliferation of cell lines.

## Conclusions

In the present study, the MS profiling of tick cell lines was applied in an attempt to clarify their nature. The quality of MS profiles was highly improved after introduction of FA/SA mixtures, allowing recognition of three distinct clusters by PCA. Cell line IRE/CTVM19 was clustered separately from the other IRE cells, and the traditional proteomic workflows proved this result. Protein profiles of acidic extracts and 2D proteomic maps of IRE11 and IRE/CTVM20 cells were more similar between each other. Comparison of MS profiles of tick cell lines and tick organs revealed several organ-specific MS signals. Five peaks in the cell lines profiles were also present in ovaries, three in the gut and salivary glands, and one in Malpighian tubules. Moreover, 11 MS signals in the cell line profiles were assigned with proteins for the first time. This work contributes to the endeavor of an efficient application of tick cell lines as a model system for the investigation of host-vector-pathogen interactions.

## Methods

### Chemicals and enzymes

α-Cyano-4-hydroxycinnamic acid (CHCA), 2,5-dihydroxybenzoic acid (DHB), ferulic acid (FA), sinapinic acid (SA), trifluoroacetic acid (TFA), Pharmalyte 3-10, urea, thiourea, CHAPS, iodoacetamide, 4-sulfophenyl isothiocyanate (SPITC), o-methylisourea bisulfate and tris-(2-carboxyethyl) phosphine were obtained from Sigma-Aldrich (Steinheim, Germany). Protein Calibration Standard I and Peptide Calibration Standard II were obtained from Bruker Daltonik (Bremen, Germany). Formic acid and dithiothreitol (DDT) were purchased from Merck (Darmstadt, Germany). Ammonium bicarbonate was obtained from Lach-Ner (Neratovice, Czech Republic). Sodium dodecyl sulfate (SDS) was obtained from Carl Roth (Karlsruhe, Germany). Organic solvents, all of analytical grade, were from various suppliers. Prestained protein Marker VI (10–245) was from AppliChem (Darmstadt, Germany).

### Tick cell lines

*Ixodes ricinus*-derived tick cell lines IRE11 [17], IRE/CTVM19 and IRE/CTVM20 [4], and *I. scapularis*-derived tick cell lines ISE6 [18] and ISE18 [19], were maintained in L15 medium supplemented with 20% bovine fetal serum, antibiotic/antimycotic mix (all Biosera, Nuaille, France) and 10% tryptose phosphate broth (Sigma-Aldrich) in 10 ml flat sided cell culture tubes (Nunc, Rochester, USA) in a cell incubator at 28 °C as described in [20]. Confluent tick cell cultures were harvested by pipetting into sterile 2 ml microtubes and spun at 600× *g* for 5 min at room temperature. The supernatant was removed and cells were resuspended in phosphate-buffered saline, pH 7.4 (PBS) and spun at 600× *g* for 5 min. The PBS washing step was repeated two more times. Upon supernatant removal, the cell pellets were stored at − 80 °C until further use.

### *Ixodes ricinus* fully-fed female dissection

Pathogen-free *I. ricinus* adult females were collected in a tick rearing facility of Institute of Parasitology AVCR. They originate from the 2nd generation of ticks collected in the Branišov forestal area of České Budějovice, Czech Republic, where transovarial pathogen transmission is not likely. Adult females were fed on laboratory guinea-pigs to full engorgement. Replete females were dissected under an SMZ-143 Stereomicroscope (Motic, Hong Kong) in a droplet of sterile PBS. Salivary glands, ovaries and Malpighian tubules were removed, washed in a droplet of PBS, and transferred to a sterile 1.5 ml microtube containing 50 μl of PBS. The midgut was removed and transferred to a sterile Petri dish filled with PBS where it was thoroughly cleaned of the host blood and transferred to a sterile 1.5 ml microtube containing 50 μl of PBS. Organs were stored at − 80 °C until further use.

### Sample preparation and MALDI-TOF MS analysis

Tick cell line pellets were resuspended in 100 μl of solution of acetonitrile (ACN)/2.5% TFA (v/v) in a volume ratio of 7:3, incubated with shaking at room temperature for 30 min, and centrifuged at 10,000× *g* for 5 min. Tick organs were ground using an MM 400 mixer mill (Retsch, Haan, Germany) in 30 μl of a mixture of ACN/2.5% TFA (v/v) in a volume ratio of 7:3, incubated with shaking on a vortex at room temperature for 30 min, and then centrifuged at 10000× *g* for 5 min. All samples were prepared in at least 3 biological replicates.

The samples were spotted onto an MSP AnchorChip™ 384 target plate (Bruker Daltonik) in triplicate. The dried droplet method was used for sample preparation whereby 0.5 μl of both samples and matrix solutions (see below) were applied to the target. Mass spectrometric measurements were performed on an Autoflex Speed MALDI-TOF/TOF (Bruker Daltonik). Mass spectra were acquired in the positive linear ion mode using pulsed extraction with an acceleration voltage of 19.5 kV, an extraction voltage of 18.2 kV, a lens voltage of 7.0 kV and a delayed extraction time of 290 ns. Resulting spectra were accumulated from up to 1000 laser shots.

Four matrices were evaluated to optimize the sample preparation procedure for MALDI-TOF MS. CHCA, DHB, FA and SA were dissolved in ACN/2.5% TFA (v/v) in a volume ratio of 7:3 at concentrations of 10 and 20 mg/ml each. Further, FA:SA mixtures with concentrations of 10:5, 5:5, 5:10 and 5:15 mg/ml, respectively, were tested.

Mass spectra processing was performed using flexAnalysis 3.4 (Bruker Daltonik). The principal component analysis (PCA) was performed using ClinProTools v.3.0 (Bruker Daltonik). mMass software was used to build average spectra and their subsequent comparison.

### One-dimensional gel electrophoresis, in-gel digestion and microgradient separation of peptides

Protein extracts of tick cell lines in ACN/2.5% TFA (v/v ratio 7:3) were dried in a speed-vac. Samples were dissolved in 0.1 M ammonium bicarbonate solution supplemented with 6 M urea. The protein concentration was measured using a BCA Protein Assay Kit (Thermo Fisher Scientific, Waltham, MA, USA).

Tricine-SDS-PAGE was carried out as described in [21]. Briefly, 10 µg of protein extracts were separated in 16% resolving gel and stained with colloidal Coomassie Brilliant Blue G-250 [22]. Gels were run in a Mini-PROTEAN II unit (Bio-Rad, Hercules, CA, USA). Gel bands of interest were cut from the gel.

Both in-gel digestion of proteins and peptide extraction from the gel were performed according to a standard protocol [23]. The extracted peptides were dissolved in 0.1% (v/v) TFA and separated using a manual microgradient device [24]. The device consists of a lab stand as a scaffold and a gas-tight microsyringe connected to a small capillary, prepared from FEP tubing, packed with 3.0 μm core-shell C18-based particles.

The microcolumn was wetted with 80% ACN/0.1% TFA and equilibrated with 0.1% TFA. After a sample loading, the microcolumn was washed with 5 μl of 0.1% TFA. Peptides were eluted using a gradient of ACN from 2 to 50% (total volume of 12 μl) supplemented with 0.1% TFA. The eluate was manually deposited on an MSP AnchorChip™ 384 target plate in 0.5 μl aliquots and mixed with 0.5 μl of CHCA matrix solution.

### Tick cell lysis and protein extraction for two-dimensional gel electrophoresis

A lysis buffer (4% SDS in 50 mM sodium-phosphate buffer pH 8.0) was added to tick cell line pellets. Cells were incubated with shaking on a vortex at room temperature for 20 min followed by incubation at 95 °C for 5 min. Then, samples were cooled down on the bench and sonicated for 10 min. Proteins were precipitated using the methanol-chloroform method [25].

### Two-dimensional gel electrophoresis

Tick cell lysate samples (~500 µg) were dissolved in 320 µl of 2D rehydration buffer (7 M urea, 2 M thiourea, 2% CHAPS, 40 mM DTT, 1% bromophenol blue and 0.2% Pharmalyte 3-10), and were used for the rehydration of 17 cm immobilized pH gradient (IPG) strips overnight. Isoelectric focusing (IEF) was performed in 17 cm IPG strips with a non-linear pH range of 3–10 (SERVA, Heidelberg, Germany) using an IEF100 (Hoefer, Holliston, MA, USA) with a maximum current of 50 mA/strip. IEF was run for a total of 40 kVh. Following IEF, the strips were reduced in equilibration buffer (6 M urea, 0.375 M Tris, pH 8.8, 2% SDS and 20% glycerol) containing 2% DTT over 15 min and then alkylated in equilibration buffer containing 2.5% iodoacetamide for 15 min.

The second dimension was performed on 12% SDS-PAGE gels (20 × 20 cm) using an OWL cell (Thermo Fisher Scientific). The resultant 2D gels were stained with colloidal Coomassie Brilliant Blue G-250 [22]. Gel images were captured with a gel documentation system, G:Box Chemi XX6 (Syngene, Cambridge, UK). Proteomic maps were analyzed using SameSpots software (TotalLab, Newcastle upon Tyne, UK). All samples were run at least in triplicate. Protein spots were excised from the gel, digested as described above, modified with *o*-methylisourea and SPTIC and identified using MALDI-TOF/TOF.

### Derivatization of tryptic peptides

A derivatization protocol of tryptic peptides with *o*-methylisourea and SPTIC was adopted from [26, 27]. The guanidination reaction mixture was prepared by mixing 5 µl of peptide digest with 5.5 µl of 7 M NH_4_OH and 1.5 µl of 5.7 M o-methylisourea bisulfate. After incubation at 65 °C for 20 min, the reaction was terminated by adding 15 µl of 10% TFA (v/v), followed by pH adjustment to the value of 8 by 7 M NH_4_OH. The sulfonation reaction was carried out at 50 °C for 1 h by mixing of guanidated peptides with 45 μl of SPITC (10 mg/ml in 20 mM NaHCO_3_). The reaction was terminated by adding formic acid to a final concentration of 5% (v/v). The acidified peptide mixture was applied onto microgradient column as described above. The modified peptides were eluted onto MSP AnchorChip™ 384 target plate in 0.5 µl steps with increasing ACN concentrations from 8 to 80% and covered with 0.5 µl of CHCA matrix.

### MALDI-MS and MS/MS analysis

MALDI-TOF/TOF experiments were performed on an Autoflex Speed mass spectrometer (Bruker Daltonics). Fragmentation spectra were acquired using the LIFT technology in the positive ion mode. The mass spectrometer was controlled by flexControl v.3.4 software for acquisition, flexAnalysis v.3.4 and ProteinScape v.3.1 for spectra processing, and WarpLC v.1.3 for automatic measurement of microgradient MALDI fractions.

Database searches were performed using the program Mascot Server v.2.4 (Matrix Science, London, UK) against the in-house prepared database containing protein sequences from ticks downloaded from NCBI database (version 20180314, 171796 sequences) supplemented with sequences of common contaminants (Max Planck Institute of Biochemistry, Martinsried, Germany). Parameters used for the database search were as follows: enzyme specificity: trypsin, two missed cleavages were allowed; fixed modifications: carbamidomethylation of cysteine; variable modifications: N-terminal protein acetylation and methionine oxidation. Precursor ion tolerance was set at 50 ppm, whereas the mass tolerance for MS/MS fragment ions was set at 0.5 Da.

### In-solution digestion of protein extracts

Protein pellets were dissolved in 0.1 M ammonium bicarbonate, containing 6 M urea. Proteins were reduced with 5 mM tris-(2-carboxyethyl) phosphine at 25 °C for 45 min and alkylated with 55 mM iodoacetamide in the dark at 25 °C for 30 min. Subsequently, the reaction mixture was diluted to a final volume of 200 μl by the addition of 50 mM ammonium bicarbonate. Then proteolysis at a protein-to-trypsin ratio of 50:1 was performed overnight. The digestion was terminated by the addition of formic acid to a final concentration of 5%. The obtained peptide mixtures were pre-fractionated and purified using C18 and cation-SR Empore^™^ disks (3M, St. Paul, USA) as described in [28].

### Liquid chromatography and mass spectrometry

The peptide analysis was carried out on a tandem mass spectrometer ESI-Q-TOF PREMIER (Waters, Milford, MA, USA) coupled to a nanoACQUITY nanocapillary liquid chromatography system (Waters). The mobile phase A consisted of 0.1% formic acid in MS grade water and mobile phase B consisted of 0.1% formic acid in 100% ACN. Purified peptides (1 µl) were loaded and washed on a Symmetry C18 Trapping column (180 µm i.d., 20 mm length, particle size 5 µm, reverse phase; Waters) with a flow rate of 5 µl/min for 3 min. After washing, the peptides were eluted from the trapping column onto an analytical column (75 µm i.d. 150 mm length, BEH300 C18, particle size 1.7 µm, reverse phase; Waters) and separated by a 40-min-long multi-step gradient at a constant flow rate of 0.4 µl/min (0 min, 3% B; 30 min, 40% B; 32 min, 85% B; 37 min, 85% B; 40 min, 3% B). Raw data were acquired in data-independent MS^E^ identity mode (Waters). Precursor ion spectra were acquired with collision energy 5 V and fragment ion spectra with collision energy 15–35 V ramp in alternating 1 s scans.

The acquired MS and MS/MS data were submitted for database searching using the PLGS v.3.0 software (Waters) against the in-house prepared database described above. Parameters used for the database search were as follows: enzyme specificity: trypsin, two missed cleavages were allowed; fixed modifications: carbamidomethylation of cysteine; variable modifications: N-terminal protein acetylation and methionine oxidation; minimum ion matches per peptide: 3; false discovery rate (FDR): 1%. Proteins were considered as significant hits if they were identified in at least two independent biological replicates.

## Additional files

**Additional file 1: Table S1.** List of all proteins identified using 1DE followed by MALDI-TOF/TOF MS/MS.

**Additional file 2: Table S2.** Lists of all proteins identified in the acidic extracts of IRE11 tick cell line using in solution digestion followed by ESI-Q-TOF-MS/MS. **Table S3.** Lists of all proteins identified in the acidic extracts of IRE/CTVM19 tick cell line using in solution digestion followed by ESI-Q-TOF-MS/MS. **Table S4.** Lists of all proteins identified in the acidic extracts of IRE/CTVM20 tick cell line using in solution digestion followed by ESI-Q-TOF-MS/MS. **Table S5.** Lists of all proteins identified in the acidic extracts of ISE6 tick cell line using in solution digestion followed by ESI-Q-TOF-MS/MS. **Table S6.** Lists of all proteins identified in the acidic extracts of ISE18 tick cell line using in solution digestion followed by ESI-Q-TOF-MS/MS.

**Additional file 3: Figure S1.** Comparison of proteins identified in the acidic extracts of tick cell lines by nanoLC-ESI-Q-TOF MS/MS. a, *I. ricinus* derived tick cell lines. b, *I. scapularis* derived tick cell lines and IRE/CTVM19.

**Additional file 4: Table S7.** List of all proteins identified using 2DE followed by MALDI-TOF/TOF MS/MS.

## Abbreviations

CHCA: α-cyano-4-hydroxycinnamic acid
DHB: 2,5-dihydroxybenzoic acid
FA: ferulic acid
SA: sinapinic acid
TFA: trifluoroacetic acid
ACN: acetonitrile
PCA: principal components analysis
