# Supplementary material for: Tissue-specific signatures in tick cell line MS profiles
Source: Parasit Vectors. 2019 May 6;12:212. doi: 10.1186/s13071-019-3460-5 (PMC6503378; doi:10.1186/s13071-019-3460-5)
Supplement: Supplementary file 1 — Additional file 1: Table S1. List of all proteins identified using 1DE followed by MALDI-TOF/TOF MS/MS. [file 13071_2019_3460_MOESM1_ESM.docx]

**Additional file 1: Table S1.** List of all proteins identified using 1DE followed by MALDI-TOF/TOF MS/MS.

| **Band^(^**^a)^ | **Accession^(b)^** | **Description^(c)^** | **Organism** | **Mw, kDa^(d)^** | **Score^(e)^** | **SC, %^(f)^** | **No. of pept.^(g)^** |
| --- | --- | --- | --- | --- | --- | --- | --- |
| **IRE11** | | | | | | | |
| 2 | V5HGP9 | putative secreted protein pk-4 precursor | *Ixodes ricinus* | 12 | 201.47 | 51.8 | 6 |
|  | A0A131Y447 | putative profilin binds actin monomers | *Ixodes ricinus* | 13.9 | 121.28 | 33.1 | 3 |
| 3 | A0A131YAE6 | putative histone h4, partial | *Ixodes ricinus* | 11.4 | 114.77 | 28.8 | 3 |
|  | A0A131Y5V6 | putative cytoplasmic cystatin, partial | *Ixodes ricinus* | 8.6 | 102.74 | 33.3 | 2 |
| 4 | V5HUZ2 | putative fatty acid-binding protein fabp | *Ixodes ricinus* | 15.4 | 127.71 | 24.4 | 3 |
|  | A0A023FHH5 | putative histone 2a *Ixodes scapularis* | *Amblyomma cajennense* | 13.3 | 124.32 | 12.9 | 2 |
| 5 | V5HKW0 | putative cyclophilin 1, partial | *Ixodes ricinus* | 20.2 | 224.63 | 31.5 | 5 |
| 6 | A0A0K8R904 | putative secreted salivary gland peptide | *Ixodes ricinus* | 21.5 | 123.23 | 23.8 | 3 |
|  | A0A0K8RL85 | putative 40s ribosomal protein s10 | *Ixodes ricinus* | 18.1 | 93 | 18.6 | 2 |
|  | Q6VPP5 | thioredoxin peroxidase, partial | *Ixodes ricinus* | 19.1 | 89.49 | 8.3 | 1 |
| 7 | A0A0K8RKT7 | putative glutathione S-transferase mu class *Rhipicephalus annulatus* glutathione S-transferase | *Ixodes ricinus* | 25.5 | 205.84 | 16.6 | 5 |
|  | A0A0K8RRH7 | putative glutathione S-transferase, partial | *Ixodes ricinus* | 24.4 | 113.34 | 12.8 | 3 |
|  | A0A131Y732 | putative thioredoxin peroxidase, partial | *Ixodes ricinus* | 24.5 | 110.71 | 19.8 | 3 |
| 8 | A0A131Y0B8 | putative nucleoside diphosphate-sugar hydrolase of the mutt nudix family | *Ixodes ricinus* | 27.5 | 267.36 | 22.8 | 5 |
| 9 | A0A0K8RS67 | putative histone, partial | *Ixodes ricinus* | 21 | 94.50 | 8.8 | 1 |
| **IRE19** | | | | | | | |
| 1 | A0A0K8RQA6 | putative ubiquitin/40s ribosomal protein s27a fusion, partial | *Ixodes ricinus* | 14.5 | 114.48 | 11.6 | 2 |
| 2 | V5HGP9 | putative secreted protein pk-4 precursor | *Ixodes ricinus* | 12 | 144.12 | 40.9 | 4 |
|  | A0A131Y5V6 | putative cytoplasmic cystatin, partial | *Ixodes ricinus* | 8.6 | 100.15 | 49.3 | 3 |
| 3 | V5IK85 | putative histone h4, partial | *Ixodes ricinus* | 11.5 | 144.89 | 38.8 | 5 |
| 4 | V5HJX3 | putative histone h2a | *Ixodes ricinus* | 13.6 | 126.6 | 18.4 | 3 |
|  | V5HKA6 | putative cytochrome b5 | *Ixodes ricinus* | 14.5 | 85.4 | 12.5 | 1 |
|  | V5HVJ7 | putative small heat shock protein ii, partial | *Ixodes ricinus* | 19.7 | 92.68 | 15.8 | 2 |
| 5 | A0A147BVR2 | putative cyclophilin type peptidyl-prolyl cis-trans isomerase, partial | *Ixodes ricinus* | 19.8 | 94.7 | 7.7 | 2 |
| 6 | V5HVJ7 | putative small heat shock protein ii, partial | *Ixodes ricinus* | 19.7 | 266.08 | 36.8 | 5 |
| 7 | A0A147BI33 | putative glutathione S-transferase | *Ixodes ricinus* | 26.3 | 160.81 | 11.8 | 3 |
| 8 | V5I1E1 | putative nucleoside diphosphate-sugar hydrolase of the mutt nudix family, partial | *Ixodes ricinus* | 25.6 | 91.99 | 6.1 | 1 |
| **IRE20** | | | | | | | |
| 1 | A0A0K8RPM6 | putative ubiquitin/40s ribosomal protein s27a fusion, partial | *Ixodes ricinus* | 9.7 | 158.81 | 36 | 3 |
| 4 | V5HKA6 | putative cytochrome b5 | *Ixodes ricinus* | 14.5 | 81.5 | 12.5 | 1 |
| 6 | V5HVJ7 | putative small heat shock protein ii, partial | *Ixodes ricinus* | 19.7 | 107.80 | 18.1 | 2 |
| **ISE6** | | | | | | | |
| 1 | A0A090X8L8 | putative polyubiquitin babesia bovis t2bo polyubiquitin, partial | *Ixodes ricinus* | 11 | 115.1 | 32 | 3 |
| 2 | B7QHT2 | profilin, putative, partial | *Ixodes scapularis* | 14.1 | 120.2 | 32.3 | 3 |
| 3 | Q4PM69 | histone H4, putative | *Ixodes scapularis* | 11.4 | 151.03 | 32 | 3 |
|  | A0A131Y5V6 | putative cytoplasmic cystatin, partial | *Ixodes ricinus* | 8.6 | 122.64 | 30.7 | 2 |
| 4 | B7QCB3 | cytochrome B5, putative | *Ixodes scapularis* | 15.1 | 193.7 | 37.3 | 3 |
|  | A0A023FHH5 | putative histone 2a *Ixodes scapularis* | *Amblyomma cajennense* | 13.3 | 99.12 | 12.9 | 2 |
|  | V5HZA1 | putative histone h2a | *Ixodes ricinus* | 13.5 | 88 | 18 | 2 |
| 5 | Q4PLY3 | cyclophilin A | *Ixodes scapularis* | 17.6 | 189.43 | 18.1 | 3 |
|  | A0A023FFH5 | putative calmodulin, partial | *Amblyomma cajennense* | 16.7 | 121.42 | 19.6 | 2 |
|  | B7PMY6 | actin depolymerizing factor, putative | *Ixodes scapularis* | 16.8 | 90.57 | 21.8 | 3 |
| 6 | B7P328 | manganese superoxide dismutase, putative, partial | *Ixodes scapularis* | 24.8 | 94.6 | 6.7 | 1 |
|  | B7Q5L2 | calponin, putative | *Ixodes scapularis* | 21 | 82.8 | 10.61 | 2 |
|  | A0A0K8RL85 | putative 40s ribosomal protein s10 | *Ixodes ricinus* | 18.1 | 81.3 | 9.6 | 2 |
| 7 | A0A0K8RKT7 | putative glutathione S-transferase mu class *Rhipicephalus annulatus* glutathione S-transferase | *Ixodes ricinus* | 25.5 | 280.64 | 16.1 | 6 |
|  | A0A147BI33 | putative glutathione S-transferase | *Ixodes ricinus* | 26.3 | 181.89 | 11.4 | 3 |
|  | B7Q8W6 | alkyl hydroperoxide reductase, thiol specific antioxidant, putative | *Ixodes scapularis* | 24.9 | 94.6 | 14.9 | 2 |
| 8 | B7QFY3 | ADP-ribose pyrophosphatase, putative | *Ixodes scapularis* | 27.5 | 226 | 17.1 | 3 |
|  | G3MQ03 | hypothetical protein | *Amblyomma maculatum* | 27.9 | 161.11 | 21 | 5 |
| 9 | B7P5X8 | voltage-dependent anion-selective channel, putative | *Ixodes scapularis* | 30.3 | 163.86 | 9.6 | 2 |
|  | A0A0K8RNH5 | putative annexin, partial | *Ixodes ricinus* | 35.2 | 114.43 | 9.7 | 2 |
| **ISE18** | | | | | | | |
| 1 | A0A090X8L8 | putative polyubiquitin babesia bovis t2bo polyubiquitin, partial | *Ixodes ricinus* | 11 | 94.8 | 13.4 | 1 |
| 2 | A0A131Y5V6 | putative cytoplasmic cystatin, partial | *Ixodes ricinus* | 8.6 | 80.35 | 30.7 | 2 |
| 3 | Q4PM69 | histone H4, putative | *Ixodes scapularis* | 11.4 | 135.63 | 31.1 | 3 |
|  | Q4PMA0 | cytoplasmic cystatin | *Ixodes scapularis* | 9 | 96.5 | 31.6 | 2 |
| 4 | B7QCB3 | cytochrome B5, putative | *Ixodes scapularis* | 15.1 | 97.44 | 11.9 | 1 |
| 5 | B7Q0J9 | cyclophilin A, putative | *Ixodes scapularis* | 21.8 | 85.1 | 13.4 | 2 |
| 6 | B7Q645 | secreted salivary gland peptide, putative, partial | *Ixodes scapularis* | 19.7 | 159.6 | 17 | 2 |
|  | B7Q5L2 | calponin, putative | *Ixodes scapularis* | 21 | 82.8 | 14.2 | 2 |
|  | A0A0K8RL85 | putative 40s ribosomal protein s10 | *Ixodes ricinus* | 18.1 | 80.4 | 9.6 | 2 |
| 7 | A0A0K8RKT7 | putative glutathione S-transferase mu class *Rhipicephalus annulatus* glutathione S-transferase | *Ixodes ricinus* | 25.5 | 100.60 | 16.1 | 3 |
| 8 | B7QFY3 | ADP-ribose pyrophosphatase, putative | *Ixodes scapularis* | 27.5 | 116.7 | 5.7 | 1 |

(a) Index number of excised band (b) This accession number refers to the sequence retrieved from the UniProt protein database. (c) A description of the respective protein accession. (d) A theoretical molecular weight. (e) The probability-based score value resulting from the MS/MS search. (f) Sequence coverage (SC) calculated from the peptide MS/MS data. (g) Number of identified peptides.
