# Supplementary figures and images for: Tissue-specific signatures in tick cell line MS profiles
Source: Parasit Vectors. 2019 May 6;12:212. doi: 10.1186/s13071-019-3460-5 (PMC6503378; doi:10.1186/s13071-019-3460-5)

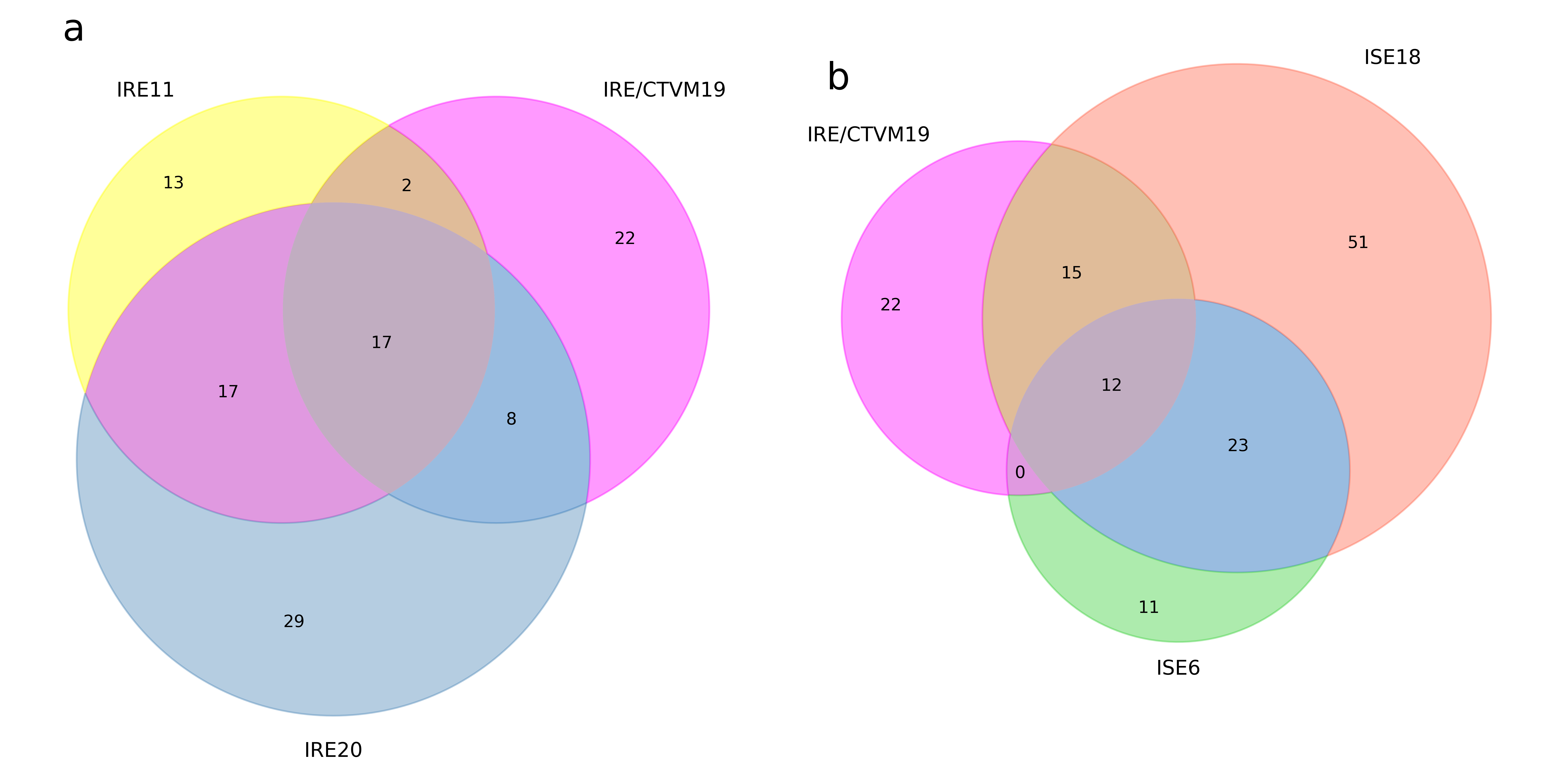

Supplement: Supplementary file 3 — Additional file 3: Figure S1. Comparison of proteins identified in the acidic extracts of tick cell lines by nanoLC-ESI-Q-TOF MS/MS. a, I. ricinus derived tick cell lines. b, I. scapularis derived tick cell lines and IRE/CTVM19. [file 13071_2019_3460_MOESM3_ESM.png]
